# Comparative analysis of patients’ survival on hemodialysis vs. peritoneal dialysis and identification of factors associated with death

**DOI:** 10.1590/2175-8239-JBN-2021-0242en

**Published:** 2022-05-04

**Authors:** Carolina Aparecida de Almeida Vicentini, Daniela Ponce

**Affiliations:** 1 Universidade Estadual Paulista, Faculdade de Medicina, Botucatu, SP, Brasil.; 2 Universidade Estadual Paulista, Faculdade de Medicina, Hospital das Clínicas, Botucatu, SP, Brasil.

**Keywords:** Survival, Death, Renal Dialysis, Peritoneal Dialysis, Sobrevida, Morte, Diálise Renal, Diálise Peritoneal

## Abstract

**Introduction:**

There are several studies comparing the outcomes of patients treated with peritoneal dialysis (PD) and hemodialysis (HD), and most are divergent.

**Methods:**

This is a cohort study that followed patients with incident PD and HD in a planned and unplanned way, in a dialysis unit of the HCFMB from 01/2014 to 01/2019, until the outcome. We collected clinical and laboratory data. The PD and HD groups, death and non-death outcomes, were compared using the chi-square test for categorical variables and t-test, or Mann-Whitney test for continuous variables. Kaplan Meier curve and log-rank test were used for survival. Multivariate analysis was performed using the Cox regression. The significant difference was p < 0.05.

**Results:**

We had 592 patients, 290 treated by HD and 302 by PD. The mean age was 59.9 ± 16.8, with a predominance of males (56.3%), the main underlying disease was diabetes (45%); 29% of the patients died. There was no difference in the survival of patients treated by HD and PD. The oldest age (1.018 (95% CI 1.000-1.037; p=0.046)) was identified as a risk factor for death, while the highest number of infection-free days (0.999 (95% CI 0.999-1.000; p=0.003 )) as a protective factor.

**Conclusion:**

The analysis reinforced that the survival of patients on HD and PD was similar. Higher age and shorter infection-free time were associated with death.

## Resumo

**Introdução:**

Há vários estudos que comparam os desfechos de pacientes tratados por diálise peritoneal (DP) e hemodiálise (HD), e a maioria é divergente.

**Métodos:**

Estudo de coorte que acompanhou pacientes incidentes em DP e HD de modo planejado e não planejado em unidade de diálise do HCFMB de 01/2014 a 01/2019, até o desfecho. Foram coletados dados clínicos e laboratoriais. Comparou-se os grupos DP e HD, óbito e não óbito, por meio do teste qui-quadrado para variáveis categóricas e teste t ou Mann-Whitney para as contínuas. Curva de Kaplan Meier e log-rank test foram utilizados para a sobrevida. A análise multivariada foi realizada por Regressão de Cox. A diferença significante foi de p < 0,05.

**Resultados:**

Foram estudados 592 pacientes, 290 tratados por HD e 302 por DP. A média de idade foi de 59,9 ± 16,8, com predomínio de sexo masculino (56,3%), principal doença de base, diabetes (45%). Óbito ocorreu em 29% dos pacientes. Não houve diferença na sobrevida de pacientes tratados por HD e DP. A maior idade (1,018 (IC 95% 1,000-1,037; p=0,046)) foi identificada como fator de risco para o óbito, enquanto a maior quantidade de dias livres de infecção (0,999 (IC 95% 0,999-1,000; p=0,003)), como fator de proteção.

**Conclusão:**

A análise reforçou que a sobrevida dos pacientes em HD e DP é semelhante. Foram associados ao óbito a maior idade e o menor tempo livre de infecção.

## Introduction

Chronic kidney disease (CKD) is considered a global public health issue and its incidence and prevalence rates are increasing. According to the Brazilian Society of Nephrology, the main causes of CKD are arterial hypertension and diabetes.^1^ Data from 2018 from the Brazilian Society of Nephrology (SBN) show that, in Brazil, hemodialysis is the most used modality of renal replacement therapy (RRT) among patients with kidney disease, 92.7%, with PD being the method used in only 7.3% of patients.^2^

RRT is highly complex and implies a high cost for public coffers - R$19.7 billion spent on RRT and R$1.2 billion with kidney transplantation.^3^ Access to both methods occurs unequally, and some countries have implemented policies to improve access to PD, which requires less infrastructure because it can be performed at home.

The possible explanation for the underutilization of PD is the fact that HD is associated with greater technological advances; fear of infection, mechanical and metabolic complications associated with PD, difficulties in inserting the peritoneal catheter and, finally, with the worst financial reimbursement in PD.^4^

Several studies have compared clinical outcomes of patients treated by PD and HD and, to date, there is no evidence of superiority of one method over the other with regard to overall mortality within the first two years of treatment.^5,6^

Some studies have reported that central venous catheters (CVC) are directly associated with lower survival, especially in the first 90 days of RRT.^5-8^ In this scenario, PD emerges as an option for unplanned RRT in patients with CKD final stage without functioning vascular access, which may offer the advantages of less use of temporary CVCs.^6^

There are several studies that analyze the factors associated with the death of patients with CKD on dialysis. In a study by the Urology and Nephrology Research Center, non-surviving hemodialysis patients had a higher rate of diabetes mellitus (56.5% vs. 34.5% in survivors, p < 0.001), had a higher prevalence of cardiac ischemia (72% vs. 29.5%, p < 0.001), were older (63.7 ± 13.2 vs. 53.8 ± 15.3 years, p < 0.001), had a higher percentage of central venous catheter (29.8% vs. 17%, p < 0.001), higher Charlson comorbidity index (3.8 ± 1.8 vs. 1.2 ± 1.2, p < 0.001) and lower serum albumin level (3.7 ± 0 .4 g/dL vs. 4.0 ± 0.3 g/d, p < 0.001) and serum creatinine (7.7 ± 2.3 mg/dL vs. 9.1 ± 2.9 mg/dL, p < 0.001).^9^

Based on the divergences of reports, more and more comprehensive studies are needed to identify factors associated with worse survival in RRT.

## Hypothesis

The survival of patients on HD and PD is similar, and the risk factors associated with lower survival are older age, having diabetes, lower levels of albumin and hemoglobin at the beginning of therapy.

## Objective

The objective of the study was to comparatively assess the survival of patients on RRT (HD vs. PD) using prediction models for censored data in order to identify the risk/protection factors that interfere with the survival of patients on dialysis.

## Methodology

This is a cohort, observational, longitudinal, retrospective and prospective study that followed up incident patients on PD and HD in a planned and urgent way, in a tertiary hospital unit from January 2014 to January 2019. Patients older than 18 years of age were included and from them we collected general identification data, such as name, sex and age, underlying disease, presence of comorbidities, dialysis start date, baseline and 6-month values of Hb, albumin, P, PTH and creatinine. Hospitalization and infection data (peritonitis, exit orifice infection, and bloodstream infection) were also collected. The patients were followed up until August 2020, and the outcomes (death, recovery of function, transplantation, change of method or transfer) were noted, in addition to survival days.

The results were analyzed and compared using a statistical program (Bioestat 5.0, IBM SPSS Statistics 20). Initially, we ran a descriptive analysis and measured central tendency and dispersion, calculated for continuous variables and frequencies (%) for categorical variables. In the PD and HD groups, we compared death and non-death using the chi-square test for categorical variables and t test - if normal distribution - or Mann-Whitney - if non-normal distribution - for continuous variables.

Death and infectious complications were defined as dependent variables. Kaplan-Meier curve and log-rank test were used to compare patient and technique survival times. Censored events were considered: death, kidney transplantation, change of method, transfer or recovery of kidney function. Cox regression was used to determine factors associated with mechanical and infectious complications, as well as patient survival. Significant variables in univariate analysis were selected for regression. The significant difference was considered for p < 0.05. The project was approved by the Research Ethics Committee (CAAE number 25875219.0.0000.5411).

## Results

During the period from August 1, 2020, to November 20, 2020, we collected data from 592 patients (290 on hemodialysis and 302 on peritoneal dialysis), who started dialysis urgently and planned, at the dialysis unit in the State of São Paulo, in the period between January 2014 and September 2019.

In the population studied, the mean age was 59.9 ± 16.8, with a predominance of males (56.3%), the main underlying disease was diabetes (46.6%), and 43.8% of the population had an episode of infection associated with the dialysis.

Table 1 shows the HD vs. PD variables and the results. Upon comparing the data, we found similarities regarding age, initial creatinine levels, initial PTH levels, number of male patients, patients with diabetes, transplanted patients who had a second episode of infection. In the HD vs. DP, statistically significant differences were found regarding a 6-month creatinine value, baseline albumin level, 6-month albumin level, baseline hemoglobin level, 6-month hemoglobin level, 6-month PTH, baseline phosphorus level, 6-month phosphorus level, 6-month Kt/V value. Regarding the number of comorbidities, HD patients had more comorbidities in relation to PD, a greater number of infection-free days - BSI data in HD patients and peritonitis data in PD patients were considered - a greater number of hospitalizations, in addition to greater survival in relation to PD. In addition, the HD group had more patients with two or more comorbidities, a higher number of hospitalized patients, a higher number of deaths, a lower number of infections. Peritonitis for PD and BSI for HD - and a lower number of patients who recovered function compared to the DP group, according to Table 1.

**Table 1:** Hd vs. dp comparative analysis

Variables	Hemodialysis (n = 290)	Peritoneal dialysis GT (n = 302)	P
Age (years)*	60.7 ± 16.4	59.0 ± 17.1	0.1
Creatinine**	6.5(4.9-8.5)	6.8(5.0-8.3)	0.3
Creatinine 6m**	8.1(6.2-10.3)	7.3(5.9-9.6)	0.01
Albumin**	3.5 (3.0-3.9)	3.4(2.8-3.8)	0.01
Albumin 6m**	3.8(3.5-4.1)	3.3(2.5-3.8)	<0.0001
PTH**	167.0 (79.3-307.8)	181.0(98.0-328.5)	0.1
PTH 6m**	206.0(112.0-351.3)	142.5(43.8-269.0)	<0.0001
Hemoglobin**	9.5(8.4-10.7)	10(8.9-11.4)	0.001
Hemoglobin 6m**	11.3(10.0-12.4)	10.9(9.3-12.1)	0.03
P**	5.7(4.6-7.1)	5.8 (4.7-7.4)	<0.0001
P 6m**	5.3(4.5-6.4)	4.8(3.8-5.7)	<0.0001
Kt/v 6 m**	0.0(0.0-1.3)	2.1(1.8-1.5)	< 0.0001
No of comorbidities**	3.0(2.0-4.0)	2.0(1.0-3.0)	0.0001
Infection-free days **	348.0 (61.0-752.0)	140(32.0-309.5)	<0.0001
No of hospital admissions**	1.0(1.0-3.0)	0.0(0.0-1.0)	<0.0001
Survival days**	483.0(148.0-1139.0)	367(111.8-677.8)	<0.0001
Males	153(52.8)	172(57.0)	0.3
Diabetic	138(47.6)	138(45.7)	0.6
2 or more comorbidities	238(82.1)	63(20.9)	<0.0001
Infection	66(22.8)	93(30.8)	0.03
Second episode of infection	26(9.0)	26(8.6)	0.9
Hospital admission	230(79.3)	88(29.1)	<0.0001
Death	108(37.2)	63(20.9)	<0.0001
Function recovery	6(2.1)	31(10.6)	<0.0001
Transplant	26(9.0)	34(11.3)	0.4

* Mean ± standard deviation,

** Median (quartiles)

The general population was broken down into death and non-death groups, and we ran a comparative analysis. These groups showed a statistically significant difference in terms of factors such as baseline creatinine levels, 6-month creatinine, age, baseline PTH level, baseline P level, 6-month Kt/V value, number of hospitalizations, number of comorbidities, number of infection-free days, survival, two or more comorbidities, number of diabetics and number of hospitalized patients. Among the comorbidities, the presence of heart failure was the most prevalent (in 75%) in patients. In addition, they showed similarities regarding baseline albumin levels, 6-month albumin level, 6-month PTH level, baseline hemoglobin levels, 6-month hemoglobin levels, 6-month P levels, being male, having had an infection - peritonitis for patients on PD and BSI for patients on HD - or a second episode of infection, as shown in Table 2.

**Table 2:** Death vs. no death comparative analysis in the general population

Variables	HD + PD Death (n = 171)	HD + PD No death (n = 421)	P
Age (years)*	67.1 ± 13.2	56.9 ± 17.2	< 0.0001
Creatinine**	5.9(4.5-7.5)	7.1(5.3-8.7)	< 0.0001
Creatinine 6m*	7.5 ± 2.6	8.2 ± 2.9	0.03
Albumin**	3.4(2.8-3.7)	3.5(3.0-3.9)	0.1
Albumin 6m**	3.6(3.2-4.0)	3.6(3.0-4.0)	0.5
PTH**	155(71.5-244.0)	189(101.0-338.0)	0.0022
PTH 6m**	190.0(92.8-298.5)	175.0(83.7-320.0)	0.4
Hemoglobin**	9.5(8.4-11.0)	9.9(8.7-11.1)	0.10
Hemoglobin 6m**	11.0(9.8-12.4)	11.1(9.9-12.3)	0.4
P**	5.2(4.1-6.6)	5.4(4.5-7.0)	0.02
P 6 months**	4.8(3.8-6.2)	4.8(4.0-6.0)	0.5
Kt/v 6m**	1.1(0.0-1.8)	1.4(0.0-2.0)	0.01
No of comorbidities*	2.9 ± 1.7	2.3 ± 1.7	0.0001
No of hospital admissions**	1.0 (1.0-2.0)	0.0 (0.0-1.0)	< 0.0001
Infection-free days**	165(35.3-350.3)	223(54.0-536.0)	0.0015
Survival days**	304.5(77.8-624.3)	466(148.0-909.0)	0.0001
Males	96(56.1)	229(46.6)	0.7
2 or more comorbidities	150(87.7)	318(75.5)	0.001
Infection	46(26.9)	113(26.8)	1.0
Second infection episode	15(8.8)	37(8.8)	1.0
Diabetic	95(55.6)	181(43.0)	0.01
Hospital admissions	127(74.3)	191(45.4)	< 0.0001

* Mean ± standard deviation,

** Median (quartiles)

The HD and PD groups were subdivided into death and non-death, and the result obtained is shown in Table 3. The comparison within the HD group, subdivided into death and non-death, found similarities regarding the initial PTH value, PTH value of 6, hemoglobin value, hemoglobin value of 6 months, initial phosphorus value, phosphorus value of 6, Kt/V value of 6 months, with respect to the patient being male and the patient having had BSI or ESI . In addition, the death group had lower initial creatinine, lower creatinine at 6 months, lower albumin at baseline, lower albumin at 6 months, older age, higher number of comorbidities, higher number of hospitalizations, lower number of BSI-free days, lower number of ESI-free days, lower survival, higher number of diabetics, higher number of comorbidities and longer hospitalization compared to the non-death group, as shown in Table 3.

**Table 3:** Death vs. no death comparative analysis of the population in hemodialysis and peritoneal dialysis

Variables	HD death (n = 108)	HD No death (n = 182)	P	PD Death (n = 63)	PD No death (n = 236)	p
Age (years)*	66.6 ± 13.6	57.3 ± 17.0	< 0.001	68.0(61.0-76.0)	59.0(45.0-70.0)	<0.0001
Creatinine**	6.1(4.6-7.6)	7.0(5.2-8.8)	0.002	5.9(4.1-7.1)	7.1(5.3-8.5)	0.0004
Creatinine 6m*	7.6 ± 2.6	8.8 ± 3.0	0.003	7.4(6.5-8.8)	7.3(5.7-9.8)	0.4
Albumin**	3.4(2.8-3.7)	3.6(3.1-3.9)	0.003	3.4 ± 0.7	3.3 ± 0.6	0.2
Albumin 6m**	3.7(3.4-4.1)	3.8(3.6-4.2)	0.005	3.2(2.5-3.7)	3.3(2.7-3.8)	0.2
PTH **	154(68.0-254.0)	182(85.6-323.0)	0.1	155.5(80.4-236.0)	189.0(111.0-365.8)	0.01
PTH 6m**	206(107.3-316.0)	211(121.8-365.3)	0.3	134.0(50.3-267.0)	145.0(43.0-269.5)	0.4
Hemoglobin**	9.3(8.4-10.9)	9.7(8.4-10.7)	0.2	10.1(8.7-11.4)	10.0(8.9-11.4)	0.5
Hemoglobin 6m*	11.1 ± 1.8	11.2 ± 2.0	0.4	10.9(9.3-12.1)	11.0(9.3-12.1)	0.4
P**	5.0(3.8-6.4)	5.0(4.2-6.4)	0.2	5.5(4.7-7.0)	5.9(4.7-7.5)	0.2
P 6m**	5.3(4.6-6.6)	5.3(4.6-6.1)	0.3	4.8(3.9-6.0)	4.8(3.7-5.7)	0.4
Kt/V 6m**	1.1(0.9-1.4)	1.2(0.9-1.3)	0.5	2.2(1.9-2.5)	2.1(1.7-2.5)	0.3
No of comorbidities *	3.4 ± 1.7	2.8 ± 1.9	0.01	2.1 ± 1.5	1.9 ± 1.4	0.2
No of hospital stays**	1.0(1.0-3.0)	1.0(0.8-2.0)	0.02	1.0(0.0-1.0)	0.0(0.0-0.0)	0.0002
BSI-free days**	235.0(52.5-482.0)	434.0(97.0-882.0)	0.0002	-	-	-
Peritonitis-free days	-	-	-	95.0(29.0-250.0)	150.0(37.0-340.0)	0.046
ESI-free days**	166(49.5-334.5)	320.5(72.3-666.3)	0.001	-	-	-
Survival days**	326(90.5-745.0)	664.5(260.5-1331.0)	< 0.001	261.0(73.0-548.0)	390.0(125.0-706.0)	0.03
Diabetic	60(55.6)	78(42.9)	0.04	35(55.6)	103(43.1)	0.1
Males	55(50.9)	98(53.9)	0.6	41(65.1)	131(54.8)	0.1
2 or more comorbidities	96(88.9)	141(77.5)	0.02	53(84.1)	177(74.1)	0.1
BSI	23(21.3)	43(23.6)	0.7	-	-	-
ESI	36(33.3)	64(35.2)	0.8	13(20.6)	56(23.4)	0.6
Peritonitis	-	-	-	23(36.5)	70(29.9)	0.3
Hospital admissions	96(88.9)	134(73.6)	0.002	31(49.2)	57(23.9)	< 0.0001

* Mean ± standard deviation,

** Median (quartiles)

The PD group was also subdivided into death and non-death, and the comparative analysis found no differences regarding 6-month creatinine, baseline albumin, 6-month albumin, 6-month PTH, baseline hemoglobin, 6-month hemoglobin, baseline phosphorus, phosphorus at 6 months, in relation to the number of comorbidities, in relation to Kt/V of 6, in relation to the sex being male, being diabetic, having two or more comorbidities, having had ESI, having had peritonitis, having had complications, surgical relocation, and planned initiation. As for the differences, the death group had older age, greater number of hospitalizations, lower initial creatinine value, lower initial PTH value, lower number of peritonitis-free days, lower survival and higher number of hospitalized in relation to the non-death group, according to Table 3.

**Figure 1:**
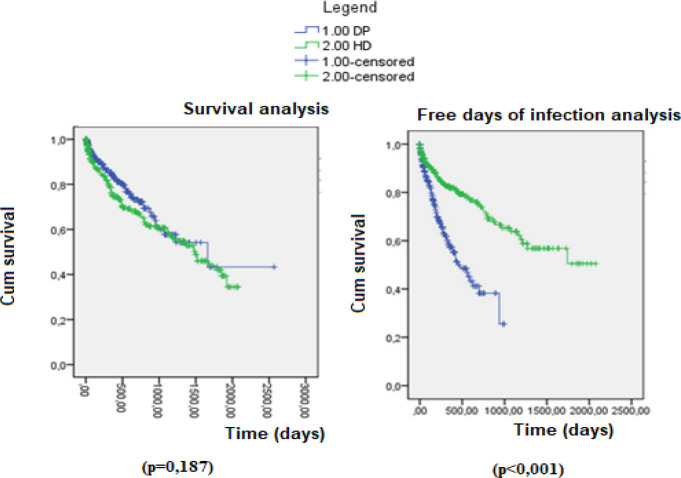
Survival and infection-free time analysis of patients in HD and PD.

The PD and HD groups were compared in terms of survival and infection-free time. There was no difference in the survival curve of patients treated with HD and PD (p=0,187). Patients treated by HD had longer infection-free time (p<0,001), as shown in Figure 1. The censored data were transplantation, change of technique, change of address and recovery of function.

The variables that in univariate analysis presented p < 0.05 were selected for the Cox Regression model. Tables 4, 5 and 6 present the COX regression for the events of death in the general population, death in the HD population and death in the PD population, respectively.

For the general dialysis population, that is, those on PD and HD, the lowest number of infection-free days (0.999 (95% CI 0.999 - 1.000; p = 0.003)), and the oldest (1.02 (95% CI) 1.00 - 1.04; p = 0.046)) were risk factors for death, as shown in Table 4. For HD patients, the presence of more than two comorbidities was also associated with death (1.2 (95% CI) 1.01 - 1.5; 0.034)), as shown in Table 5. For patients on PD, older age (1.04 (95% CI 1.02 - 1.07; p = 0.04)) and lower number of infection-free days (0.997 (95% CI 0.996 - 0.999; p = 0.002)) were also risk factors for death, as shown in Table 6.

**Table 4:** Cox-regression for death in the general population in dialysis (hd and pd)

	p	HR (Hazard ratio)	95.0% CI for HR	
			Lower	Upper
Creatinine 6m	0.7	1.0	0.9	1.1
No of Hospital admissions	0.8	1.0	0.9	1.2
Hospital admissions	0.1	1.9	0.9	3.8
Diabetic	0.7	0.9	0.5	1.5
Comorbidities	0.1	1.1	1.0	1.3
Infection-free days	0.003	0.999	0.999	1.0
Kt/V 6m	0.8	1.0	0.8	1.2
Age	0.046	1.02	1.00	1.04
Creatinine	0.7	1.0	0.9	1.1
Phosphorus	1.0	1.0	0.9	1.0
Initial PTH	0.6	1.0	1.0	1.0

**Table 5:** Cox-regression for death in the hemodialysis population

	p	HR (Hazard ratio)	95.0% CI for HR	
			Lower	Upper
Albumin 6m	0.3	1.0	1.0	1.04
No of hospital admissions	0.6	1.0	0.9	1.2
Hospital admissions	0.6	1.3	0.5	3.3
Age	0.3	1.010	0.991	1.030
Creatinine	0.8	0.999	0.989	1.009
Creatinine 6m	0.4	0.996	0.988	1.005
Albumin	0.3	0.986	0.961	1.012
ESI-free days	0.2	1.000	0.999	1.000
BSI-free days	0.000	0.999	0.998	0.999
Comorbidities	0.034	1.2	1.01	1.5
Diabetic	0.9	0.9	0.5	1.7

**Table 6:** Cox regression for death in the peritoneal dialysis population

	p	HR (Hazard ratio)	95.0% CI for HR	
			Lower	Upper
Age	0.04	1.04	1.02	1.07
Initial creatinine	0.6	0.979	0.90	1.07
Initial PTH	0.2	0.999	0.998	1.000
Hospital admission	0.6	0.998	0.992	1.005
Peritonitis-free days	0.002	0.997	0.996	0.999

## Discussion

Several studies have compared clinical outcomes of patients treated by PD and HD and, to date, there is no evidence of superiority of one method over the other with regards to overall mortality within the first two years of treatment. When analyzing subpopulations, some studies have shown better results with PD in the group of young patients without comorbidities, while other studies show lower mortality after two years of dialysis in elderly patients with comorbidities treated with HD.^4,6,10^

In this study, the univariate analysis showed that the mortality of patients on HD was higher than the mortality of patients on PD, but the HD population had a greater number of comorbidities. When the time to death factor was considered, there was no difference in the survival of patients on HD and PD. In fact, the result of the comparative analysis of survival in the present study corroborates the initial hypothesis that the survival of patients on HD and PD is similar.

The most common underlying diseases among dialysis patients were diabetes and hypertension, similar to that described in the literature, in addition to a large number of undetermined underlying diseases, especially in PD patients, which can be explained by the lack of pre-dialysis follow-up in many of them.

Univariate analysis showed that diabetes, older age, shorter time free of infection and comorbidities, and lower levels of albumin and hemoglobin would be associated with death in the dialysis population. Among the comorbidities, heart failure was the most prevalent, in 75% of the patients. However, after performing the Cox regression, only the variables age, time free of infection and more than two comorbidities were associated with death.

Our data reinforce the evidence that survival declines with increasing age, but do not corroborate previous results that point to diabetes, hypoalbuminemia, inadequacy of dialysis, anemia and higher PTH levels as predictors of mortality.^10,11^

Acchiardo et al.^11^ found that malnutrition (represented by low albumin) is the most significant predictor of death on dialysis. The USRDS (United States Renal Data System) found a linear relationship of decreased mortality with increasing dialysis dose (Kt/V from 0.8 to 1.4),^12^ while HEMO, a prospective randomized clinical trial, did not detect improvements in survival of patients whose dialysis dose was higher (Kt/V = 1.65), compared with those with a conventional dose (Kt/V = 1.25).^13^ Therefore, it is possible that very high doses of Kt/ V do not contribute to better survival. Anemia is a frequent and early complication of CKD and contributes to cardiovascular changes in patients with CKD, mainly promoting left ventricular hypertrophy and association with death.^14,15^

Among the factors involved in mineral metabolism, different PTH values directly interfere with the survival of patients on dialysis, by increasing the risk of cardiovascular death, since it is associated with the acceleration in the formation of atherosclerosis and arterial calcifications.^16^

Collinearity with age or with the presence of multiple comorbidities may be an explanation for these variables not remaining significant in the multivariate analysis.

In the present study, the highest number of comorbidities was associated with death in HD, both in univariate and multivariate analyses. The population analyzed in this study had a more advanced average age, and it is possible to compare the results obtained here with those from a study focused on the analysis of mortality in the elderly on dialysis, which relates diabetes and longer dialysis time to mortality in the elderly.^17^ Among the comorbidities, heart failure was the most prevalent, affecting 75% of the patients.

Through multivariate analysis, it is possible to see that greater age and fewer days free of infection are risk factors for death in the general population (PD + HD) and in PD. In HD, the lowest number of infection-free days and the highest number of comorbidities are risk factors.

The study’s limitations were the statistical analysis itself, which, for example, relates the number of hospitalizations to a greater occurrence of infections, when, in fact, it is the infection itself that will be the cause of these patients’ hospitalizations; and data on mechanical complications related to dialysis accesses and the presence of residual renal function during therapies were also not evaluated. In addition, the planned or unplanned initiation of HD and hemodialysis access, factors that may impact the prognosis of patients, were not analyzed.

As for comorbidities, it may be necessary, in future studies, to specify them, in order to know which ones are directly related to death. Finally, it is important to emphasize that the tertiary hospital, which hosted the study, exceptionally has a greater number of patients on PD than on HD,^12,13^ because PD is the preferred dialysis therapy at the beginning, due to the lack of vacancy for HD and that PD has the advantage of not requiring a CVC - a catheter associated with lower survival.^18-20^

## Conclusions

The survival of patients on HD and PD was similar, according to the initial hypothesis. The following were associated with death: older age, shorter infection-free time and number of comorbidities.

## References

[1] Penico MG, Paulo Koah (2016). Cartilha dia Mundial do Rim.

[2] (2017). Censo da Sociedade Brasileira de Nefrologia.

[3] Beraldo N. (2019). Saúde alerta para prevenção e diagnóstico precoce da Doença Renal Crônica.

[4] Niang A; A, Iyengar A, Luychx V. (2018). Hemodialysis versus peritoneal dialysis in resource-limited setting. Current Opinion in Nephrology and Hypertension.

[5] Thiery A, Séverac F, Hannedouche T, Couchoud C, Do VH, Tiple A (2018). Survival advantage of planned haemodialysis over peritoneal dialysis: A cohort study. Nephrol Dial Transplant.

[6] Heaf JG, Lokkegaard H, Madsen M. (2002). Initial survival advantage of peritoneal dialysis relative to hemodialysis. Nephrol Dial Transplant.

[7] de Abreu MM, Walker DR, Sesso RC (2013). A cost evaluation of peritoneal dialysis and hemodialysis in the treatment of end-stage renal disease in Sao Paulo, Brazil. Perit Dial Int.

[8] Genovesi S, Porcu L, Luise MC, Riva H, Nava E, Contaldo G (2017). Sudden Death in End Stage Renal Disease: Comparing Hemodialysis versus Peritoneal Dialysis. Blood Purif.

[9] Soleymanian T, Niyazi H, Dehkordi SNJ, Savaj S, Argani H, Najafi I. (2017). Predictors of clinical outcomes in hemodialysis patients: A multicenter observational study. Iran J Kidney Dis.

[10] United States Renal Data System (2015). USRDS Annual Data Report: Epidemiology of Kidney Disease in the United States.

[11] Acchiardo SR, Moore LW, Latour PA. (1983). Malnutrition as the main factor in morbidity and mortality of hemodialysis patients. Kidney Int Suppl.

[12] Held PJ, Port FK, Wolfe RA, Stannard DC, Carroll CE, Daugirdas JT (1996). The dose of hemodialysis and patient mortality. Kidney Int.

[13] Allon M, Depner TA, Radeva M, Bailey J, Beddhu S, Butterly D (2003). Impact of dialysis dose and membrane on infection-related hospitalization and death: Results of the HEMO study. J Am Soc Nephrol.

[14] Locatelli F, Pisoni RL, Akizawa T, Cruz JM, DeOreo PB, Lameire NH, Held PJ. (2004). Anemia in hemodialysis patients of five European Countries: association with morbidity and mortality in the Dialysis Outcomes and Practice Patterns Study (DOPPS). Nephrol Dial Transplant.

[15] Pisoni RL, Bragg-Gresham JL, Young EW, Akizawa T, Asano Y, Locatelli F (2004). Anemia management and outcomes from 12 countries in the Dialysis Outcomes and Practice Patterns Study (DOPPS). Am J Kidney Dis.

[16] Steingrimsdottir Le (2005). Relationship between serum parathyroid hormone levels, vitamin D sufficiency, and calcium intake. JAMA.

[17] Han SS, Park JY, Kang S, Kim KH, Ryu DR, Kim H (2015). Dialysis modality and mortality in the elderly: A meta-analysis. Clin J Am Soc Nephrol.

[18] Bittencourt Dias D, Mendes ML, Alves CA, Caramori JT, Ponce D. (2020). Peritoneal Dialysis as an Urgent-Start Option for Incident Patients on Chronic Renal Replacement Therapy: World Experience and Review of Literature. Blood Purification.

[19] Dias DB, Mendes ML, Caramori JT, Falbo dos Reis P, Ponce D. (2020). Urgent-start dialysis: Comparison of complications and outcomes between peritoneal dialysis and haemodialysis. Perit Dial Int.

[20] Perl J, Wald R, McFarlane P, Bargman JM, Vonesh E, Na Y (2011). Hemodialysis vascular access modifies the association between dialysis modality and survival. J Am Soc Nephrol.

